# Distribution of D-3-aminoisobutyrate-pyruvate aminotransferase in the rat brain

**DOI:** 10.1186/1471-2202-15-53

**Published:** 2014-04-27

**Authors:** Masao Abe, Shinichiro Ochi, Yoko Mori, Kiyohiro Yamazaki, Takashi Ishimaru, Yuta Yoshino, Ryuji Fukuhara, Satoshi Tanimukai, Seiji Matsuda, Shu-ichi Ueno

**Affiliations:** 1 Department of Neuropsychiatry, Ehime University Graduate School of Medicine, Shitsukawa, Toon, Ehime 791-0295, Japan; 2 Department of Anatomy and Embryology, Neuroscience, Ehime University Graduate School of Medicine, Shitsukawa, Toon, Ehime 791-0295, Japan; 3 Department of Neuropsychiatry, Faculty of Medical and Pharmaceutical Sciences, Kumamoto University, 1-1-1, Honjo, Chuou-ku, Kumamoto, Kumamoto 860-8556, Japan

**Keywords:** D-3-aminoisobutyrate-pyruvate aminotransferase, Asymmetric dimethylarginine, RT-PCR, Western blotting, Immunohistochemistry

## Abstract

**Background:**

D-3-aminoisobutyrate, an intermediary product of thymine, is converted to 2-methyl-3-oxopropanoate using pyruvate as an amino acceptor by D-3-aminoisobutyrate-pyruvate aminotransferase (D-AIB AT; EC 2.6.1.40). A large amount of D-AIB AT is distributed in the kidney and liver; however, small amounts are found in the brain. Recently, D-AIB AT was reported to metabolize asymmetric dimethylarginine (ADMA) *in vivo* and was suggested to be an important enzyme for nitric oxide metabolism because ADMA is a competitive inhibitor for nitric oxide synthase. In this study, we examined the distribution of D-AIB AT in the rat brain further to understand its role. We measured D-AIB AT mRNA and protein expression using quantitative RT-PCR and Western blotting, and monitored its distribution using immunohistochemical staining.

**Results:**

D-AIB AT was distributed throughout the brain, with high expression in the cortex and hippocampus. Immunohistochemical staining revealed that D-AIB AT was highly expressed in the retrosplenial cortex and in hippocampal neurons.

**Conclusion:**

Our results suggest that D-AIB AT is distributed in the examined- just the regions and may play an important role there.

## Background

**Figure 1:**
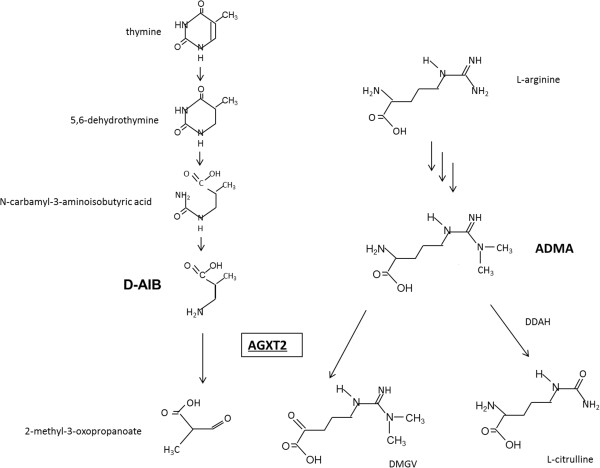
**Metabolic pathway of D**-**AIB and ADMA.** D-AIB AT can transaminate both D-AIB and ADMA with pyruvate as an amino acceptor. Abbreviations: D-AIB AT; D-3-aminoisobutyrate aminotransferase, DDAH; dimethylarginine dimethylaminohydrolase, ADMA; asymmetric dimethylarginine, DMGV; α-keto- δ-(N^G^,N^G^- dimethyl-guanidino) valeric acid. Protein names are underlined.

D-3-aminoisobutyrate-pyruvate aminotransferase (D-AIB AT, R-3-amino-2-methylpropionate-pyruvate aminotransferase, EC 2.6.1.40, alanine: glyoxylate aminotransferase 2, EC 2.6.1.44) is a unique aminotransferase that metabolizes not L- but the D-isomer of 3-aminoisobutyrate (AIB) as an amino donor and pyruvate as an amino acceptor to generate 2-methyl-3-oxopropanoate and alanine. We, as well as others, have previously purified and characterized D-AIB AT in the rat liver [1,2], and its gene structure was determined by Matsui-Lee et al. [3]. The condition resulting in high amounts of D-AIB excretion in the urine is inherited in an autosomal recessive fashion [4], and individuals with this condition have been reported to lack D-AIB AT activity [5]. D-AIB is an intermediate product of thymine, and D-AIB AT is the only enzyme that metabolizes D-AIB (Kakimoto et al. [5,6]). High amounts of D-AIB AT are distributed in the kidney and liver, and small amounts have been identified in the brain. However, no functional reason for D-AIB AT expression in the central nervous system has been identified [7]. D-AIB AT is also known to be a dimethylarginine-pyruvate aminotransferase ([8], see Figure 1), which metabolizes asymmetric dimethylarginine (ADMA) *in vivo*[9]. Therefore, it appears that D-AIB AT is also an important enzyme for nitric oxide metabolism because ADMA is a competitive inhibitor of the nitric oxide synthase (NOS) enzyme family [10]. Endothelial (eNOS) and neuronal (nNOS) play an obligatory role in the regulation of cerebral blood flow and cell viability, and in the protection of nerve cells or fibers against pathogenic factors associated with Alzheimer’s disease, Huntington’s disease, seizures, and migraines [11]. Therefore, in this study, we monitored the mRNA and protein expression of D-AIB AT in the rat brain.

## Methods

### Experimental animals

Male Wistar rats (CLEA Japan, Tokyo, Japan), 6–8 weeks old, were housed in air-conditioned rooms (temperature, 22 ± 2°C) with a 12-h light–dark cycle. After rats were anaesthetized with 30 ml of diethyl ether and decapitated, the brain was removed and tissues used for examination (frontal cortex, temporal cortex, cerebellum striatum, thalamus, hippocampus, midbrain, pons, and olfactory bulb) were dissected. All experiments were approved by the Ethics Review Committee for Animal Experimentation of Ehime University.

### RNA extraction and quantitation of D-AIB AT mRNA expression

We examined ten rat (the ages of rat breakdown was that 6 weeks old is six, 7 weeks old is two and 8 weeks old is two) for RT-PCR. RNA was extracted from each brain region isolated according to the RNeasy Lipid Tissue Mini kit instructions (Qiagen, Valencia, CA, USA), which included DNase treatment (Qiagen). Following assessment of RNA quality and quantity with the NanoDrop (NanoDrop Technologies, DE, USA), 1 μg of total RNA was used for cDNA synthesis with random hexamers using Moloney murine leukemia virus reverse transcriptase (Applied Biosystems, Austin, TX, USA). The expression of the D-AIB AT gene transcript was quantified by real-time PCR with the TaqMan Gene Expression Assay (Applied Biosystems). TaqMan primer-probe sets for D-AIB AT (agxt2, Rn00582928_m1) and glyceraldehyde-3-phosphate dehydrogenase (GAPDH, Ss03375435_u1), used as an endogenous control, were purchased from Applied Biosystems. Quantitative RT-PCR was performed using the StepOnePlus System (Applied Biosystems). Changes in D-AIB AT mRNA expression were calculated after normalization with GAPDH expression. The cDNA from an arbitrarily selected control rat were used as a calibrator sample. The ΔΔCT method provided a relative quantification ratio according to the calibrator, which allowed statistical comparisons of gene expression among samples. Values of fold changes in the sample versus the frontal cortex samples represented averages from triplicate measurements. Statistical calculations were carried out using the SPSS Statistical Software Package 11.5 (SPSS, Tokyo, Japan). The mRNA expression differences among brain tissues were analyzed by analysis of variance with repeated measures followed by the Bonferroni post hoc test. A *P* value less than 0.05 was considered statistically significant.

### Western blotting

We examined four rat (all ages of rat is 6 weeks old) for Western blotting. Brain tissue was homogenized in lysis buffer containing 8.1 mM Na_2_HPO_4_, 2.68 mM KCl, 1.47 mM KH_2_PO_4_, 137 mM NaCl, 1 mM EDTA, and 2 mM 2-mercaptoethanol. Homogenates were centrifuged at 16,000 g for 20 min at 4°C. The supernatant was decanted into a new centrifuge tube. Protein concentrations for each brain region were determined using the BCA Protein Assay Kit (Thermo Fisher Scientific K.K., Yokohama, Japan). Protein samples (10 μg) were suspended in Laemmli sample buffer, and sodium dodecyl sulfate (SDS) polyacrylamide gel electrophoresis was performed according to standard procedures. Total lysates were separated on 1% SDS-polyacrylamide gels, and were blotted onto polyvinylidene difluoride membranes (Millipore, Bedford, MA, USA). After blocking with Blocking One (Nacalai Tesque, Kyoto, Japan), the membranes were incubated with either anti-D-AIB AT rabbit polyclonal primary antibody (specificity of antibody show Additional files 1 and 2) generated using the D-AIB-AT peptide ‘SPYTLGLTNVGIYKMEL’ (Japan Bioserum, Hiroshima, Japan), or anti-GAPDH rabbit primary antibody (internal control; Abcam, Cambridge, UK) diluted in 1% (v/v) Tween 20 at room temperature for 3 h. Membranes were treated with anti-rabbit goat IgG horseradish peroxidase-linked Whole Antibody (GE Healthcare, Buckinghamshire, UK) diluted in 1% (v/v) Tween 20 at room temperature for 1 h. Visualization using ChemiDoc MP (Bio Rad) was used for detection. Image J (http://rsb.info.nih.gov/ij/index.html) was used for Western blot analysis following digitization.

### D-AIB AT immunohistochemistry

Wistar rat brains on postnatal day 7, 14, 28, and 56 were used in this study. Animals were anesthetized by intraperitoneal injection of chloral hydrate (10 mg/kg) and then perfused transcardially with physiological saline, followed by 4% (w/v) paraformaldehyde in 100 mM phosphate buffer (pH 7.4). Brains were removed and post-fixed in the same solution for 12 h at 4°C and embedded in paraffin wax. Frontal sections of rat brains were cut at 5 μm, dewaxed in xylene, and rehydrated with a graded series of ethanol solutions. Histochemistry was performed using the anti-D-AIB AT antibody (this antibody same as Western) in combination with the avidin-biotin peroxidase complex (ABC) method using the VECTASTAIN ABC kit (Vector Labs, Burlingame, CA, USA). Endogenous peroxidase was blocked in methanolic hydrogen peroxidase at 36°C for 30 min, and non-specific protein binding was suppressed by incubation with 1% (w/v) bovine serum albumin at 36°C for 30 min, followed by rinsing with 0.02 M phosphate-buffered saline (PBS; pH 7.4). Sections were incubated with the anti D-AIB AT antibody at 4°C overnight. After rinsing with PBS, the sections were further incubated with the VECTASTAIN ABC reagent at room temperature for 30 min. Finally, the sections were colorized with PBS containing 0.006% (w/v) 3-3-diaminobenzidine tetrahydrochloride and 0.003% (v/v) H_2_O_2_ at room temperature for 30 min, and lightly counterstained with hematoxylin.

## Results

**Figure 2:**
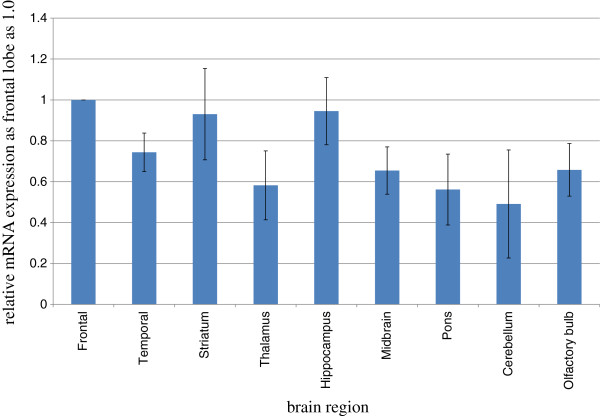
**Distribution of D**-**AIB AT mRNA in the rat brain.** The mRNA expression in the frontal cortices was used as standard and represented as 1.0 (n = 10). Bar shows the average ± S.E. Abbreviations: frontal, frontal cortex; temporal, temporal cortex.

D-AIB AT mRNA expression in the rat brain compared with GAPDH mRNA expression, used as an internal standard, is shown in Figure 2. D-AIB AT mRNA was ubiquitously distributed in rat brain tissues. The mRNA expression in the brain cortices, striatum and hippocampus were relatively high when compared with other brain regions but there were no significant differences in expression levels.

**Figure 3:**
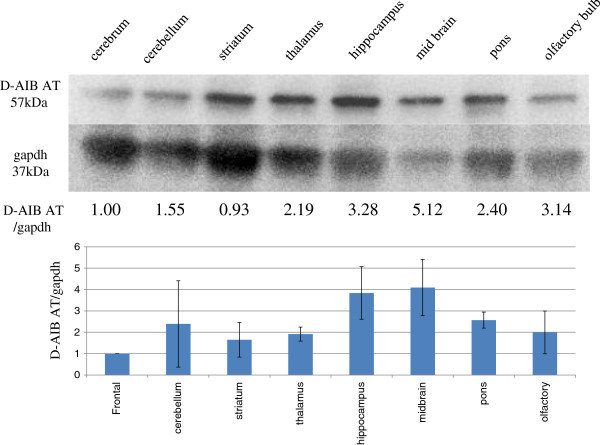
**Distribution of D**-**AIB AT protein in the rat brain with Western blotting.** Typical photograph of Western blotting of D-AIB AT and GAPDH are indicated in the upper figure. In the lower figure, density of D-AIB AT in each brain region was standardized by its GAPDH expression (n = 4).

D-AIB AT protein levels were measured by Western blotting (Figure 3). Protein levels of D-AIB AT in various brain regions were compared with GAPDH. Expression levels in parts of the midbrain (about five fold), hippocampus and olfactory bulb (about three fold) were high when compared with frontal cortex.

**Figure 4:**
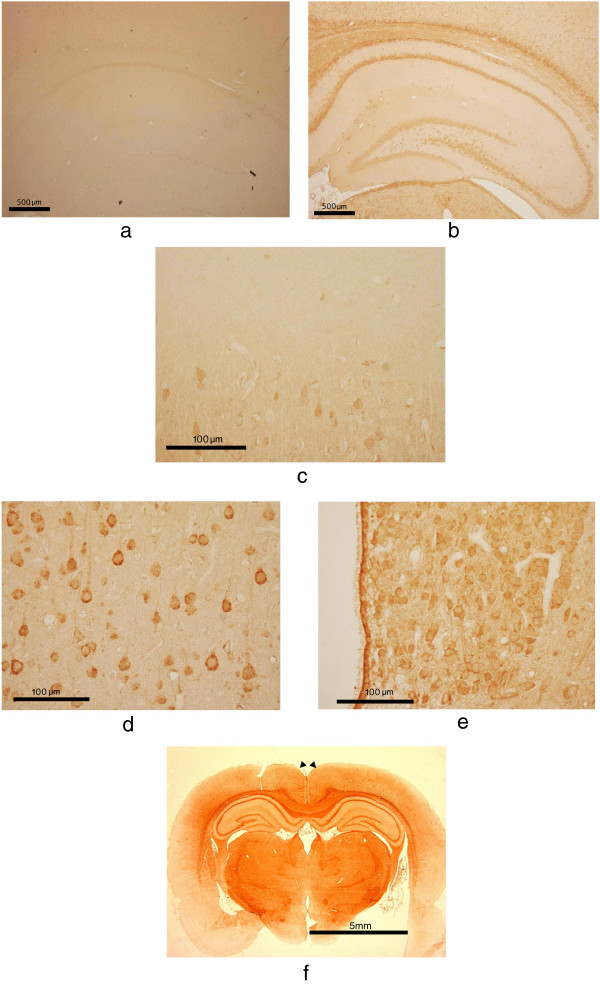
**D**-**AIB AT immunohistochemistry in 8**-**week male rat brains.** Figure 4**a** High magnification of hippocampal region with pre-immune rabbit serum, Figure 4**b**; with rabbit anti D-AIB AT antibody are shown. Higher magnification of outer layer of cerebral cortex (Figure 4**c**), inner layer of the cerebral cortex (Figure 4**d**), and brain stem (Figure 4**e**) are shown with anti D-AIB antibody. Figure 4**f**. Whole rat brains with coronal sections (arrowheads indicates retrosplenial cortex) are shown with anti D-AIB antibody.

**Figure 5:**
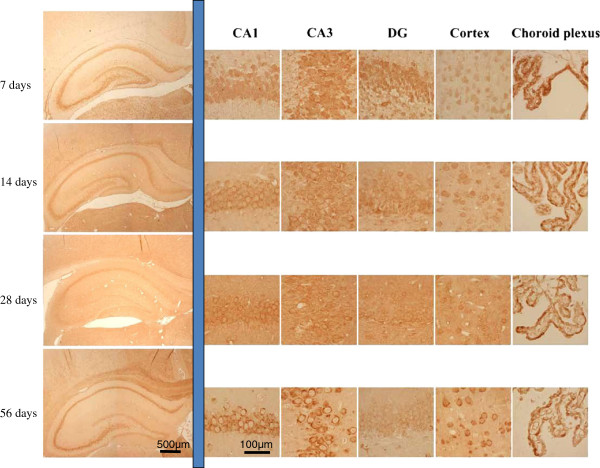
**D-AIB AT immunohistochemistry in various brain regions from 7-, 14-, 28-, and 56-days-old male rats.** The figures are: From left to right; whole figure of hippocampus, CA1, CA3, DG (dentate gyrus), cortex, and choroid plexus, From top to bottom; 7-, 14-, 28-, and 56-days postpartum male rat brains.

Immunohistochemical studies revealed that hippocampus is clearly stained with rabbit anti D-AIB AT antibody (Figure 4b, compared with Figure 4a; pre-immune rabbit serum). In the hippocampus, neurons in the CA1, CA3, CA4, and dentate gyrus were strongly stained, particularly the cytosolic region. The expression in the cerebral cortex was weaker in the outer layer (Figure 4c) than that in deep layers (Figure 4d). Neurons of the brainstem were also strongly stained (Figure 4e). Compared with other cortical regions, the retrosplenial cortex was strongly stained (Figure 4f). The rat which we examined about each brain tissues is 8 weeks old. During development (Figure 5; day 7 to 56 post partum), D-AIB AT protein was highly expressed in the hippocampus, cortex, and choroid plexus at all ages.

## Discussion

To our knowledge, this is the first report to examine the distribution of D-AIB AT in the rat brain. D-AIB AT was distributed in examined- just the regions, with expression being greatest in deep layers of the cortex, hippocampus, and brain stem. Immunohistochemistry revealed that D-AIB AT expression was highest in the retrosplenial cortex, hippocampus, brain stem, and choroid plexus.

High amounts of D-AIB AT are distributed in the kidney and liver [5], and small amounts are observed in various regions of the brain [7]. Although the only known role of D-AIB AT relates to the metabolism of D-AIB, its precise function in the central nervous system remains unclear. Recently, D-AIB AT was identified as an enzyme that not only catalyzes the metabolism of D-AIB but also catalyzes the degradation of ADMA, which is a competitive inhibitor of NOS [12]. ADMA is present in the central nervous system [13] and is reported to be ubiquitously distributed in various regions of the brain [14]. D-AIB AT inhibits NO production *in vitro*[10] and actively degrades ADMA *in vivo*[9]; however, ADMA is also degraded by dimethylarginine dimethylaminohydrolase (DDAH) 1, a member of the DDAH enzyme family [15]. We hypothesize that D-AIB AT in the brain effectively metabolizes ADMA as an ADMA: pyruvate aminotransferase, an aminotransferase that catalyzes ADMA with pyruvate to form α-keto- δ-(N^G^,N^G^- dimethyl-guanidino) valeric acid and alanine *in vivo* (Figure 1), resulting in a decrease to ADMA brain levels, which helps NOS improve endothelial NO production in the brain.

In this study, we report that D-AIB AT is distributed in the examined- just the regions. Several studies support the idea that ADMA may be important regulator of the NO system, where elevated ADMA concentrations are associated with hypertension [16], congestive heart failure [17], progression of chronic kidney disease [18] and atherosclerosis [19]. D-AIB AT may be related to the vulnerability for cerebro-vascular disease.

Our immunohistochemical studies revealed that D-AIB AT is widely expressed in the retrosplenial cortex and hippocampus. The rat retrosplenial cortex is similar to Brodmann areas 29 and 30 in primates, which has abundant reciprocal projections with the hippocampus directly and indirectly [20]. Inactivation of the retrosplenial cortex is reported to impair active navigation in dark testing conditions in a rat model with tetracaine anesthetization of the retrosplenial cortex, suggesting that this region is important for spatial memory [21]. Hippocampal atrophy is known to be one of the main symptoms of Alzheimer’s disease (AD), and disruption of fronto-hippocampal connections, not only directly but indirectly through damage of the retrosplenial posterior cingulate cortex, is observed in AD [22]. Metabolic decline in the retrosplenial cortex is also reported in AD following positron emission tomography [23,24]. Pathological changes in this region can also occur in schizophrenia, bipolar disorder, and post-traumatic stress disorder review [25]. Therefore the expression of D-AIB AT in brain lesions formed following neurological disease progression.

## Conclusion

In conclusion, D-AIB AT is widely distributed in the brain and may work as an aminotransferase not only related to degradation of D-AIB from thymine. Further studies will be needed to clarify the role of D-AIB in the central nervous system.

## Abbreviations

D-AIB: D-3-aminoisobutyrate; AT: Aminotransferase; ADMA: Asymmetric dimethylarginine; RT-PCR: Real time-polymerase chain reaction; NOS: Nitric oxide synthase; eNOS: Endothelial nitric oxide; nNOS: Neuronal nitric oxide; GAPDH: Glyceraldehyde-3-phosphate dehydrogenase; EDTA: Ethylenediaminetetraacetic acid; SDS: Sodium dodecyl sulfate; ABC: Avidin-biotin peroxidase complex; PBS: Phosphate-buffered saline; DDAH: Dimethylarginine dimethylaminohydrolase; NO: Nitric oxide; AD: Alzheimer’s disease.

## Competing interests

The authors declare that they have no competing interests.

## Authors’ contributions

MA, SO, YM, KY, TI, YY carried out the molecular genetic studies, participated in the mRNA expression and Western blotting. SM participated in the immunohistochemistry. MA have been involved in drafting the manuscript, while RF, ST, SM revised it critically for important intellectual content. SU supervised this study, participated in its design and coordination, and revised the manuscript that led to the final approval of the current submission. All authors read and approved the final manuscript.

## Supplementary Material

Additional file 1 **The ELISA data of rabbit anti D-AIB AT antibody.** The titer of the antibody was assessed by enzyme-linked immunosorbent assay. ◆; pre-immune rabbit serum, ■; anti-D-AIB AT antibody. Click here for file

Additional file 2 Western blot of rat brain tissues indicates the comparison between rabbit pre-immune serum (left; × 1000) and anti D-AIB antibody (right; × 1000). Click here for file

## References

[B1] Ueno S Morino H Sano A Kakimoto Y Purification and characterization of D-3-aminoisobutyrate-pyruvate aminotransferase from rat liver Biochim Biophys Acta 1990 15 169 175 10.1016/0304-4165(90)90008-K 2306461

[B2] Tamaki N Kaneko M Mizota C Kikugawa M Fujimoto S Purification, characterization and inhibition of D-3-aminoisobutyrate aminotransferase from the rat liver Eur J Biochem 1990 15 39 45 10.1111/j.1432-1033.1990.tb15457.x 2158891

[B3] Matsui-Lee IS Muragaki Y Ideguchi T Hase T Tsuji M Oschima A Okuno E Molecular cloning and sequencing of a cDNA encoding alanine-glyoxylate aminotransferase 2 from rat kidney Biochem 1995 15 856 862 10.1093/oxfordjournals.jbchem.a124787 7592550

[B4] Yanai J Kakimoto Y Tsujio T Sano I Genetic study of beta-aminoisobutyric acid excretion by Japanese Am J Hum Genet 1969 15 115 132 5814159 PMC1706433

[B5] Kakimoto Y Taniguchi K Sano I D-β-aminoisobutyrate:pyruvate aminotransferase in mammalian liver and excretion ob b aminoisobutyrate in man J Biol Chem 1969 15 336 340 5773299

[B6] Kakimoto Y Armstrong MD The preparation and isolation of D-(−)-beta-aminoisobutyric acid J Biol Chem 1961 15 3283 3286 14453202

[B7] Yanai I Benjamin H Shmoish M Chalifa-Caspi V Shklar M Ophir R Bar-Even A Horn-Saban S Safran M Domany E Lancet D Shmueli O Genome-wide midrange transcription profiles reveal expression level relationships in human tissue specification Bioinformatics 2005 15 5 650 659 10.1093/bioinformatics/bti042 15388519

[B8] Ogawa T Kimoto M Sasaoka K Dimethylarginine:pyruvate aminotransferase in rats. Purification, properties, and identity with alanine:glyoxylate aminotransferase 2 J Biol Chem 1990 15 20938 20945 2123486

[B9] Martens-Lobenhoffer J Rodionov RN Drust A Bode-Böger SM Detection and quantification of α-keto-δ-(N (G), N (G)-dimethylguanidino) valeric acid: a metabolite of asymmetric dimethylarginine Anal Biochem 2011 15 234 240 10.1016/j.ab.2011.08.044 21945966

[B10] Rodionov RN Murry DJ Vaulman SF Stevens JW Lentz SR Human Alanine-Glyoxylate Aminotransferase 2 Lowers Asymmetric Dimethylarginine and Protects from Inhibition of Nitric Oxide Production J Biol Chem 2010 15 5385 5391 10.1074/jbc.M109.091280 20018850 PMC2820767

[B11] Toda N Ayajiki K Okamura T Cerebral blood flow regulation by nitric oxide in neurological disorders Can J Physiol Pharmacol 2009 15 581 594 10.1139/Y09-048 19767882

[B12] Scriver CR Perry TL Scriver CR, Beaudet AL, Sly WS, Valle D Disorders of omega-amino acids in free and peptide-linked forms The Metabolic Basis of Inherited Disease Vol. 1 1989 New York: McGraw-Hill Press pp 755 771

[B13] Kotani K Ueno S Sano A Kakimoto Y Isolation and identification of methylarginines from bovine brain J Neurochem 1992 15 1127 1129 10.1111/j.1471-4159.1992.tb09371.x 1737987

[B14] Ueno S Sano A Kotani K Kondoh K Kakimoto Y Distribution of free methylarginines in rat tissues and in the bovine brain J Neurochem 1992 15 2012 2016 1431891 10.1111/j.1471-4159.1992.tb10088.x

[B15] Leiper JM Santa Maria J Chubb A MacAllister RJ Charles IG Whitley GS Vallance P Identification of two human dimethylarginine dimethylaminohydrolases with distinct tissue distributions and homology with microbial arginine deiminases Biochem J 1999 15 209 214 10.1042/0264-6021:3430209 10493931 PMC1220543

[B16] Kayrak M Bacaksiz A Vatankulu MA Ayhan SS Taner A Unlü A Yazici M Ulgen MS Association between exaggerated blood pressure response to exercise and serum asymmetric dimethylarginine levels Circ J 2010 15 1135 1141 10.1253/circj.CJ-09-0419 20453387

[B17] Wilson Tang WH Tong W Shrestha K Wang Z Levison BS Delfraino B Hu B Troughton RW Klein AL Hazen SL Differential effects of arginine methylation on diastolic dysfunction and disease progression in patients with chronic systolic heart failure Eur Heart J 2008 15 2506 2513 10.1093/eurheartj/ehn360 18687662 PMC2567021

[B18] Vallance P Leone A Calver A Collier J Moncada S Accumulation of an endogenous inhibitor of nitric oxide synthesis in chronic renal failure Lancet 1992 15 572 575 10.1016/0140-6736(92)90865-Z 1347093

[B19] Leiper J Nandi M Torondel B Murray-Rust J Malaki M O'Hara B Rossiter S Anthony S Madhani M Selwood D Smith C Wojciak-Stothard B Rudiger A Stidwill R McDonald NQ Vallance P Disruption of methylarginine metabolism impairs vascular homeostasis Nat Med 2007 15 198 203 10.1038/nm1543 17273169

[B20] Vogt BA Vogt LJ Farber NB Paxinos G Cingulate cortex and models of disease The Rat Nervous System 2004 3 U.S.A: Elservier pp705 727

[B21] Cooper BG Mizumori SJY Retrosplenial cortex inactivation selectively impairs navigation in darkness Neuro Rep 1999 15 625 630 10.1097/00001756-199902250-00033 10208601

[B22] Pengas G Williams G Cabronero JA Ash TJ Hong Y Garcia DI Fryer T Hodges J Nestor J The relationship of topographical memory performance to regional neurodegeneration in Alzheimer’s disease Front Aging Neurosci 2012 15 article 17 10.3389/fnagi.2012.00017 PMC3389330 22783190

[B23] Minoshima S Giordani B Berent S Frey KA Foster NL Kuhl DE Metabolic reduction in the posterior cingulate cortex in very early Alzheimer's disease Ann Neurol 1997 15 85 94 10.1002/ana.410420114 9225689

[B24] Villain N Desgranges B Viader F de la Sayette V MVzenge F Landeau B Baron JC Eustache FC Eust G Relationships between hippocampal atrophy, white matter disruption, and gray matter hypometabolism in Alzheimer's disease J Neurosci 2008 15 6174 6181 10.1523/JNEUROSCI.1392-08.2008 18550759 PMC2902815

[B25] Vann SD Aggleton JP Maguire EA What does the retrosplenial cortex do? Nat Rev Neurosci 2009 15 11 792 802 10.1038/nrn2733 19812579

