# Supplementary material for: Distribution of D-3-aminoisobutyrate-pyruvate aminotransferase in the rat brain
Source: BMC Neurosci. 2014 Apr 27;15:53. doi: 10.1186/1471-2202-15-53 (PMC4030283; doi:10.1186/1471-2202-15-53)
Supplement: Additional file 1 — The ELISA data of rabbit anti D-AIB AT antibody. The titer of the antibody was assessed by enzyme-linked immunosorbent assay. ◆; pre-immune rabbit serum, ■; anti-D-AIB AT antibody. [file 1471-2202-15-53-S1.pptx]

## Slide 1
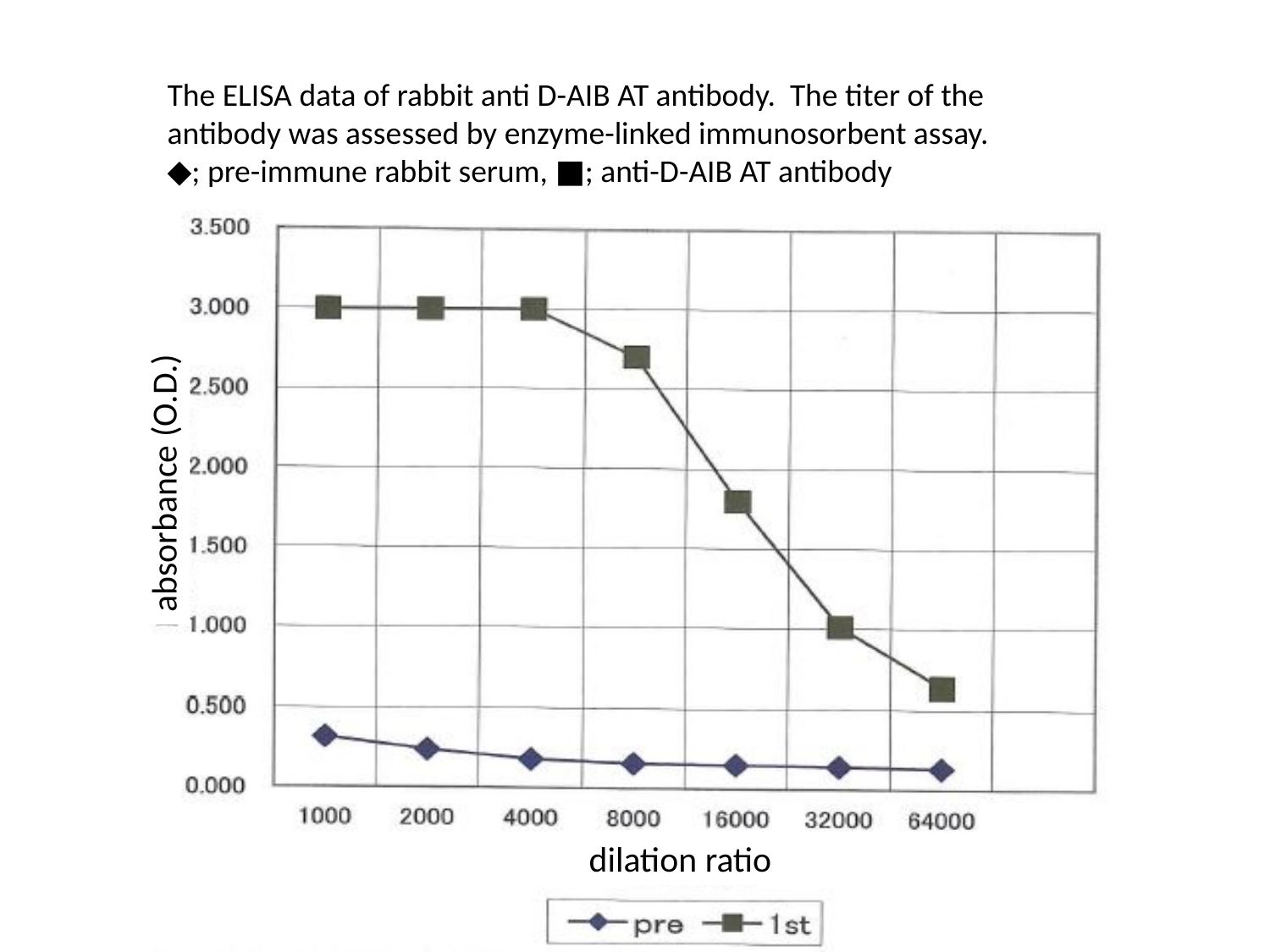

The ELISA data of rabbit anti D-AIB AT antibody. The titer of the antibody was assessed by enzyme-linked immunosorbent assay.
◆; pre-immune rabbit serum, ■; anti-D-AIB AT antibody
absorbance (O.D.)
dilation ratio
