# Supplementary material for: Distribution of D-3-aminoisobutyrate-pyruvate aminotransferase in the rat brain
Source: BMC Neurosci. 2014 Apr 27;15:53. doi: 10.1186/1471-2202-15-53 (PMC4030283; doi:10.1186/1471-2202-15-53)
Supplement: Additional file 2 — Western blot of rat brain tissues indicates the comparison between rabbit pre-immune serum (left; × 1000) and anti D-AIB antibody (right; × 1000). [file 1471-2202-15-53-S2.pptx]

## Slide 1
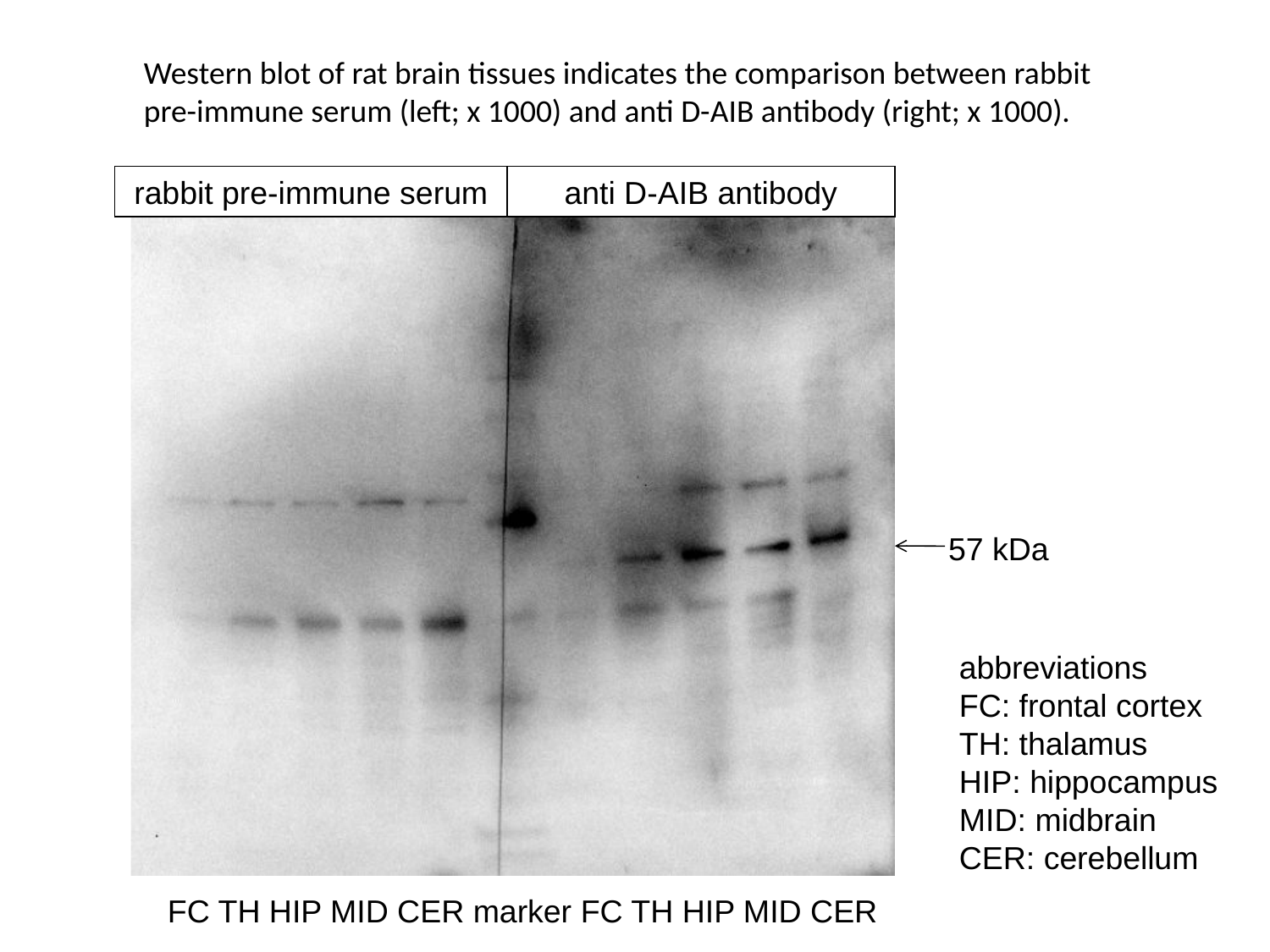

Western blot of rat brain tissues indicates the comparison between rabbit pre-immune serum (left; x 1000) and anti D-AIB antibody (right; x 1000).
rabbit pre-immune serum
anti D-AIB antibody
57 kDa
abbreviations
FC: frontal cortex
TH: thalamus
HIP: hippocampus
MID: midbrain
CER: cerebellum
FC TH HIP MID CER marker FC TH HIP MID CER
